# Applications of Bioinformatics in Cancer

**DOI:** 10.3390/cancers11111630

**Published:** 2019-10-24

**Authors:** Chad Brenner

**Affiliations:** 1 Department of Otolaryngology–Head and Neck Surgery, Michigan Otolaryngology and Translational Oncology Laboratory, University of Michigan Health Systems, Ann Arbor, MI 48109-0602, USA; chadbren@umich.edu; Tel.: +1-734-763-2761; 2 Department of Pharmacology, Michigan Otolaryngology and Translational Oncology Laboratory, University of Michigan Health Systems, Ann Arbor, MI 48109-0602, USA; 3 Rogel Cancer Center, University of Michigan Medical School, 1150 E. Medical Center Dr., 9301B MSRB3, Ann Arbor, MI 48109-0602, USA

**Keywords:** bioinformatics, machine learning, artificial intelligence, Network Analysis, single-cell sequencing, circulating tumor DNA (ctDNA), Neoantigen Prediction, precision medicine, Computational Immunology

This series of 25 articles (22 original articles, 3 reviews) is presented by international leaders in bioinformatics and biostatistics. This original series of articles details emerging approaches that leverage artificial intelligence and machine learning algorithms to improve the utility of bioinformatics applications in cancer biology. Importantly, the issue also addresses the limitations of current approaches to analyzing high throughput datasets by providing support for novel methods that can be used to improve complex multi-variable analysis. For example, in order to help identify clinically meaningful genes, Shen et al. demonstrate how the implementation of a knockoff procedure can control false discovery rates in next-generation datasets with relatively small sample sizes [1]. Additionally, tools were developed and validated to address complex problems ranging from tumor heterogeneity to mutation signature analysis. For example, intertumor heterogeneity scores were characterized from >2800 tumors and used to identify genes associated with high heterogeneity including histone methyltransferase *SETD2* and DNA methyltransferase *DNMT3A*, which were then validated by CRISPR/CAS9 in experimental systems [2]. Likewise, a tool was derived to infer tumor RNA expression signatures of genes with copy loss to support gene-loss driven biomarker analysis [3], and, a weight-matrix based approach was used to highlight the distribution of APOBEC and AID-related gene signatures in multiple cancers that drive subsets of the somatic mutation spectra [4]. Together these manuscripts demonstrate how novel tools and statistical approaches are being used to refine analysis of large next generation sequencing datasets. Extending these concepts, Veronesi et al. also develop an R-script based tool box for efficient analysis of gene signatures with diagnostic and prognostic variable that highlights how tools are being rapidly adapted into easy-to-use application packages [5].

Several papers in this series also demonstrate the potential to integrate large and diverse datasets and use machine learning approaches to develop significantly improved multi-variable predictors of clinical outcome. For example, deep learning artificial intelligence-based approaches were shown to be highly effective at integrating genomic data from multiple sources using de-noising auto-encoders to curate deep features associated with breast cancer clinical characteristics and outcomes [6]. Moreover, artificial intelligence-driven classification techniques were also used on multiple independent colorectal cancer datasets to identify and verify biomarkers of diagnosis and prognosis that may have important implications for the disease [7]. As another example, the Taiwan Cancer Registry database was analyzed to evaluate the value of the Wu co-morbidity score for accuracy in assessing curative-surgery-related 90-day mortality risk and overall survival in patients with locoregionally advanced head and neck cancer [8]; and, in an alternative approach, Ferroni et al. demonstrate the utility of using machine learning-driven decision support systems to extract data from electronic health records and refine prognostic variables [9]. As an alternative approach, and to understand how gene sets may correlate with outcome, Locati et al. utilized self-organizing map approaches to curate publicly available HPV+ cancer data and inferred gene signatures associated with three biological subtypes of the disease [10]. Novel datasets comparing the molecular composition of primary colorectal cancer and brain metastases were also generated [11]. In an interesting informatics approach, analysis of steroid hormone-related gene sets in publicly available data identified steroidogenic acute regulatory protein as a potential prognostic biomarker in breast cancer [12]. Likewise, a meta-analysis of GEO and TCGA miRNA datasets led to the prioritization of candidate biomarkers of prognosis and overall survival in oral cancer [13]. Machine learning approaches were similarly used to prioritize relevant miRNAs and validate the high performance of highly ranked miRNAs in classification models, suggesting that prioritization of targets from expression data is a highly effective strategy [14]. Analysis of miRNA data using an observed survival interval was reported to overcome issues with clinical outcome associations [15]. Collectively suggesting the potential of these approaches in this new era of machine learning approaches. Finally, additional analysis of similar datasets also highlighted the role of detailed characterization of clinical characteristics in avoiding biological and the clinical outcome analysis bias in large dataset analysis was well demonstrated in the analysis of pancreatic cancer TCGA data by Nicolle et al. [16]. 

More broadly, machine learning-driven informatics approaches, which were demonstrated to have utility in improving statistical analysis of integrated histopathologic datasets, were implemented to analyze the TCGA lung adenocarcinoma dataset as an alternative approach to modeling outcomes [17]. Furthermore, using both the lung adenocarcinoma and hepatocellular carcinoma datasets to analyze the utility of integrated gene and imaging data, multiple individual genes, conditional on imaging features, were shown to drive significant improvement in prognosis modeling [18]. These improvements in integrated multi-feature image analysis and molecular analysis for outcome modeling suggest that complex models incorporating diverse variables may be key to making substantial improvements to clinical outcome models in the future.

Interestingly, several of the articles also highlight the ability to use emerging bioinformatic techniques, high throughput small molecule screening data, and/or outcomes data to make improved predictive models. Lu et al. leveraged a support vector machine learning algorithm to analyze datasets from the Cancer Cell Line Encyclopedia and identify a 10-gene predictive model of recurrence-free survival and overall survival in epithelial ovarian cancer, validated on two independent datasets [19]. Diverse bioinformatics approaches were used to demonstrate how Bufadienolide-like chemicals may contribute to cardiotoxicity and function as anti-neoplastic agents providing a roadmap for prioritizing the mechanisms of action of small molecules with recent informatics techniques [20]. Further, a novel pipeline was developed to predict acquired resistance to EGFR inhibition, in which the team built a meta-analysis-based, multivariate model that leveraged eight independent studies and had high predictive performance [21]. Network pharmacologic analysis was used as an approach to nominate herb-derived compounds for their potential efficacy in tumor immune microenvironment regulation and tumor prevention [22], showing the utility of informatics approaches for deconvolution of drug screening data. 

The collection also includes insightful reviews discussing major bioinformatics approaches involved in the analysis of cell-free DNA sequencing data for detecting genetic mutation, copy number alteration, methylation change, and nucleosome positioning variation [23]; how bioinformatics approaches can be used to understand the functional effects of *TERT* regulation by alternative splicing [24]; and how automatic computer-assisted methods and artificial intelligence-based approaches may be leveraged for brain cancer characterization in a machine and deep learning paradigm [25].

The diversity of approaches and datasets highlighted in this collection of articles underscore the broad range of bioinformatics techniques that are being developed to answer complex questions ranging from how to better predict clinical outcomes to prioritizing lead compounds capable of disrupting the tumor-immune microenvironment. The articles collectively demonstrating the machine learning approaches can be used to make significant advances in cancer biology. Indeed, as we develop a better understanding of how different machine learning approaches are best suited to pursue critical questions as outlined in the articles of this series, we can ultimately hope to improve research efficiency and make substantial improvements to the overall health of patients.
